# Evaluation of Primary Oral Vancomycin Prophylaxis Against *Clostridioides difficile* Infection During Autologous Stem Cell Transplantation

**DOI:** 10.1093/ofid/ofae622

**Published:** 2024-10-14

**Authors:** Michael J Williams, Sol Atienza, Erin Franzen, Heena Rathod, Brittany Mejaki, Justin Graff, Sandra Korman, Noah Zouine, Zartash Gul, Sherjeel Sana, Stephen Medlin, Brian P Buggy

**Affiliations:** Malignant Hematology and Stem Cell Transplant, Aurora St Luke's Medical Center, Milwaukee, Wisconsin, USA; Department of Pharmacy, Aurora St Luke's Medical Center, Milwaukee, Wisconsin, USA; Department of Pharmacy, Aurora St Luke's Medical Center, Milwaukee, Wisconsin, USA; Department of Pharmacy, Aurora St Luke's Medical Center, Milwaukee, Wisconsin, USA; Department of Pharmacy, Aurora St Luke's Medical Center, Milwaukee, Wisconsin, USA; Malignant Hematology and Stem Cell Transplant, Aurora St Luke's Medical Center, Milwaukee, Wisconsin, USA; Department of Pharmacy, Aurora St Luke's Medical Center, Milwaukee, Wisconsin, USA; Malignant Hematology and Stem Cell Transplant, Aurora St Luke's Medical Center, Milwaukee, Wisconsin, USA; Department of Pharmacy, Aurora St Luke's Medical Center, Milwaukee, Wisconsin, USA; Quality Management, Aurora St Luke's Medical Center, Milwaukee, Wisconsin, USA; Quality Management, Aurora St Luke's Medical Center, Milwaukee, Wisconsin, USA; Malignant Hematology and Stem Cell Transplant, Aurora St Luke's Medical Center, Milwaukee, Wisconsin, USA; Malignant Hematology and Stem Cell Transplant, Aurora St Luke's Medical Center, Milwaukee, Wisconsin, USA; Malignant Hematology and Stem Cell Transplant, Aurora St Luke's Medical Center, Milwaukee, Wisconsin, USA; Infectious Diseases Section, Aurora St Luke's Medical Center, Milwaukee, Wisconsin, USA

**Keywords:** autologous stem cell transplantation, *Clostridioides difficile* infection, oral vancomycin prophylaxis

## Abstract

**Background:**

Evaluations of oral vancomycin prophylaxis (OVP) against *Clostridioides difficile* have been reported in stem cell transplant populations with short follow-up periods. The longest known duration of standardized follow-up post-OVP is 90 days within an allogeneic stem cell transplant population. In 2017, we implemented OVP 125 mg twice daily in autologous stem cell transplant (ASCT) recipients beginning the day of admission and continued until the day of discharge.

**Methods:**

Patients who received an ASCT within our institution between 1 January 2012 and 31 December 2021 were included and separated into 2 groups based on the receipt of OVP. The primary study aim was to measure the incidence of *C difficile* infection (CDI) during the ASCT admission. A secondary aim was to evaluate for delayed CDI 180 days post-discharge. Other factors evaluated were prior history of CDI, use of systemic antimicrobials, and length of stay.

**Results:**

Overall, 254 patients were evaluated and 58% received OVP, predominantly as primary prophylaxis (95%). Of the 18 patients who developed in-hospital CDI, 6 were in the OVP group versus 12 in the non-OVP cohort (4% vs 11%, *P* = .03). In the 180-day follow-up period, OVP use did not increase risk of developing CDI after discontinuation while in-hospital length of stay was identified as a significant factor.

**Conclusions:**

The use of OVP significantly reduced the incidence of CDI during the in-hospital ASCT course without increasing CDI post-OVP use. These encouraging results should promote further research into the use of OVP in ASCT.

Clinically evident *Clostridioides difficile* infection (CDI) prevention and management remain a clinical challenge, particularly among high-risk groups such as hematopoietic stem cell transplant recipients. CDI in this population has been reported at a prevalence of 5%–30% [1–5] with recurrence rates between 35% and 50% [1, 4]. The most significant risk factor for the development of CDI is antibiotic use, which is often universal in the context of febrile neutropenia prophylaxis and subsequent treatment [1]. One approach to mitigate CDI incidence has been to use oral vancomycin prophylaxis (OVP).

A systematic review investigated the use of anti–*C difficile* antibiotics and incidence of CDI. Of the 13 trials included, only 3 specifically evaluated the stem cell transplant population and none investigated the use of OVP as primary prophylaxis in autologous stem cell transplant (ASCT) patients [6]. More recently, Shestovska et al [2] studied primary OVP in a mixed allogeneic/autologous transplant patient population and determined efficacy of OVP in reduction of CDI rates overall and shorter lengths of stay for autologous patients. As secondary prophylaxis, Ganetsky et al [3] retrospectively studied OVP in allogeneic stem cell transplants and showed 0% incidence in patients receiving OVP compared with 20% in those who did not. Their follow-up period was 90 days in which a post-OVP CDI rate of 4.4% was identified at a median of 60 days after OVP discontinuation. Morrisette and colleagues [4] reported on OVP as secondary prevention in both autologous and allogeneic stem cell transplant recipients and found a lower incidence of recurrent CDI in patients who received OVP. While use of OVP appears to reduce incidence of CDI, none of these studies assessed patients for clinical episodes of CDI for longer than 90 days after OVP cessation.

Clinicians could expect low rates of CDI when administering a preventive anti–*C difficile* agent. There is concern, however, for CDI in the post-antibiotic discontinuation period secondary to alterations in the intestinal microbiome. Microbiota research in healthy subjects has revealed that gut dysbiosis may be prolonged for up to 1 year after discontinuation of systemic antibiotics [7]. Among patients treated for CDI, Abujamel et al [8] identified an approximate 21-day “vulnerable period” where patients may be at highest risk for post-antibiotic CDI. This was defined as the time between completion of oral vancomycin to the regrowth of *C difficile–*suppressing microbiota. In another study, the most likely time for CDI recurrence was early, within 2 months of oral vancomycin cessation, with day 10 identified as bearing the greatest recurrence risk [9]. Long-term follow-up of CDI development or recurrence post-OVP in ASCT patients has not yet been well described.

OVP was implemented by our program in January 2017 in an effort to reduce CDI during the admission for ASCT. Oral vancomycin solution 125 mg twice daily was given to all ASCT patients beginning the day of inpatient conditioning chemotherapy admission and continuing until the day of discharge following count recovery. In this retrospective analysis, we aim to compare the CDI incidence during ASCT admission and for 180 days following discharge between patients who were or were not given OVP.

## METHODS

### Patient Selection and Evaluation

Patients were retrospectively identified via internal administrative records for those who received an ASCT within our community hospital for the indication of multiple myeloma or lymphoma between 1 January 2012 and 31 December 2021. Patients were excluded if they had received a stem cell transplant prior to the study window or >1 stem cell transplant in the study period. Patients were also excluded if they received an allogeneic stem cell transplant or chimeric antigen receptor T-cell therapy at any time. Testing for *C difficile* colonization at baseline was not performed, nor was routine testing for vancomycin-resistant *Enterococcus* species (VRE) at baseline or subsequently.

All patients were evaluated on the following criteria extracted from the electronic medical record (EMR): age at time of ASCT, sex, transplant indication, conditioning regimen, use of OVP, number of OVP doses received, days of systemic antibiotic use, and CDI incidence between discharge and 180 days later. The number of OVP doses was defined as the quantity of prophylactic oral vancomycin doses a patient received during the admission. Days of systemic antibiotic use consisted of inpatient calendar days within which any spectrum of antibacterial was received regardless of intent as prophylaxis or treatment. Oral vancomycin days as prophylaxis or treatment were not counted as a “systemic antibiotic.” Levofloxacin was the institutional standard prophylaxis agent for all patients in the absence of a fluoroquinolone allergy, in which case an alternative was used. Absolute neutrophil count (ANC) at time of CDI test positivity was recorded. To consistently compare follow-up data across both groups, the post-ASCT period was defined as the day of discharge through the subsequent 180 days.

Programmatic changes during the study period included the implementation of OVP at our institution on 1 January 2017. Additionally, revised local standards at the start of 2019 recommended *Pneumocystis jirovecii* pneumonia (PJP) prophylaxis beginning day +30 or at discharge, whichever occurred sooner. This consisted of either 3 times weekly oral sulfamethoxazole-trimethoprim or monthly intravenous pentamidine. From 2012 through 2018, antibacterial prophylaxis was used only during the neutropenic period. In 2019, subsequent to multisite standardization, antibacterial prophylaxis was changed to begin earlier on the day of cell infusion and continue until neutrophil count recovery.

*Clostridioides difficile* stool sample testing prior to 2018 was performed by our institutional laboratory using a qualitative test for polymerase chain reaction (PCR) detection of the cytotoxin B gene (Xpert *C difficile*; Cepheid, Sunnyvale, California). In 2018, reflex detection of the *C difficile* glutamate dehydrogenase antigen and toxins A and B (*C. DIFF QUICK CHEK COMPLETE*, Alere North America, Orlando, Florida) was added to any PCR-positive specimen. Prior to 2018, a positive PCR without additional testing was interpreted clinically as a positive CDI. Subsequently, with further enzyme immunoassay (EIA) methods, a positive EIA result signified CDI. Conversely, a negative EIA suggested colonization only, but management was left to clinical discretion. Throughout our study, *C difficile* testing followed an EMR and nurse-driven institutional protocol, which encouraged testing if a patient experienced new-onset diarrhea (>3 loose stools in a 24-hour period).

### Objectives

The primary outcome was to determine and compare the incidence of CDI (defined as the presence of new-onset diarrhea as defined above and a positive *C difficile* PCR test during the ASCT admission) between patients who were or were not given OVP. Secondary objectives were to compare the CDI incidence between groups in the post-discharge 180-day follow-up period and differences in ASCT admission length of stay.

### Statistical Analysis

Baseline characteristics were evaluated using χ^2^ or Fisher exact test for discrete values, and Mann-Whitney *U* test was used for continuous variables. A Cox proportional hazards model was created to examine the primary objective. The secondary objective of CDI incidence post-discharge was analyzed using a competing-risk Cox proportional hazards model. A 2-tailed *P* value <.05 was considered significant for all values. All statistical analysis was performed using SAS version 9.4 software.

## RESULTS

### Baseline Characteristics

Patient query yielded a total of 347 inpatient autologous transplant episodes, of which nearly one-third were patients who received >1 ASCT. Therefore, once exclusion criteria were assessed, 254 unique patients remained for analysis. Table 1 summarizes the baseline characteristics. The population's median age was 64 years (range, 20–83 years) with no statistical difference between groups (*P* = .48). The predominant transplant indication was multiple myeloma (n = 153), followed by lymphoma (n = 101). In general, conditioning regimens utilized were single-agent melphalan for myeloma and BEAM (carmustine, etoposide, cytarabine, melphalan) or carmustine plus thiotepa for lymphoma. There was no difference between groups in terms of history of CDI diagnosis pre-ASCT (2% no OVP vs 5% OVP, *P* = .23).

**Table 1.** Patient Characteristics

Characteristic	Overall (n = 254)	No OVP (n = 106)	OVP (n = 148)	*P* Value
Sex, No. (%)				.**04**
Female	122 (48)	43 (41)	79 (53)	
Male	132 (52)	63 (59)	69 (47)	
Age, y, median (range)	64 (20–83)	65 (22–83)	62.5 (20–78)	.48
Transplant indication, No. (%)				**<**.**0001**
Lymphoma	101 (40)	57 (54)	44 (30)	
Multiple myeloma	153 (60)	49 (46)	104 (70)	
Conditioning regimen, No. (%)				**<**.**0001**
BEAM	94 (37)	57 (53)	37 (25)	
Melphalan 200 mg/m^2^	130 (51)	47 (44)	83 (56)	
Melphalan 140 mg/m^2^	21 (8)	2 (2)	19 (13)	
Other^a^	9 (4)	0 (0)	9 (6)	
History of CDI, No. (%)	9 (3.5)	2 (2)	7 (5)	.23
Time from CDI to admission, d, median (range)^b^	531 (33–2143)	749 (679–819)	324 (33–2143)	.64

Significant *P* values are displayed in bold type.

Abbreviations: BEAM, carmustine, etoposide, cytarabine, melphalan; CDI, *Clostridioides difficile* infection; OVP, oral vancomycin prophylaxis.

^a^Other regimens included carmustine plus thiotepa or nonstandard, reduced-dose variations of BEAM (carmustine omitted or replaced with thiotepa) or melphalan.

^b^Evaluation of 8 patients. One additional patient had documented year of CDI but no exact date.

### Clinical Outcomes

**Figure 1.**
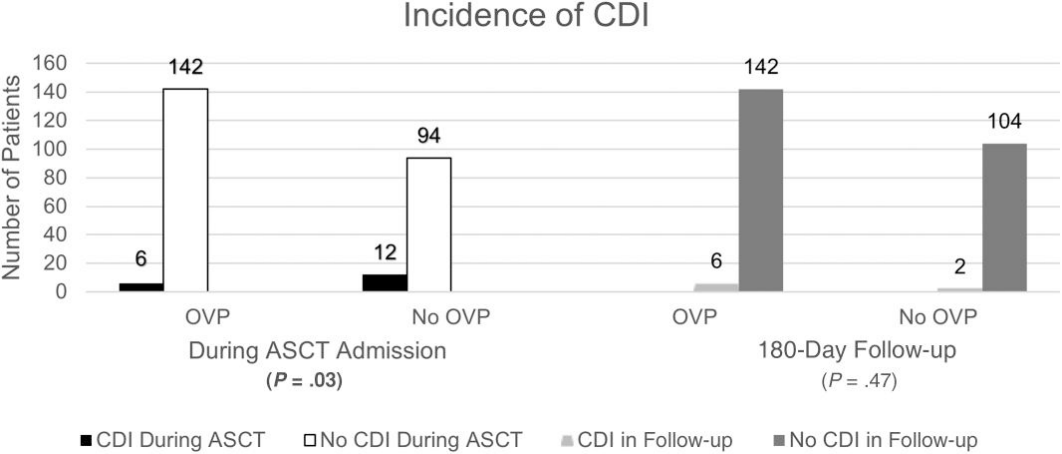
Incidence of *Clostridioides difficile* infection (CDI) during autologous stem cell transplant (ASCT) admission and 180-day post-discharge follow-up period between the oral vancomycin prophylaxis (OVP) and no-OVP groups. Significant *P* value is displayed in bold type.

One hundred six (42%) patients did not receive OVP while 148 patients (58%) did. Of these, 95% received OVP as primary prophylaxis. The overall incidence of CDI during ASCT admission and the 180-day follow-up period was 10.2%: 14 patients in the non-OVP group and 12 in the OVP cohort (13.2% vs 8.1%, respectively, *P* = .19; Table 2). CDI during the ASCT admission, also referred to as in-hospital CDI, was lower in the OVP group (4% vs 11%, *P* = .03) as shown in Figure 1. All in-hospital CDI cases were among patients receiving either BEAM or melphalan 200 mg/m^2^ conditioning regimens. Adherence to OVP from admission to the point of CDI detection was 100% among the 6 patients who were positive and 98.8% among those without CDI.

**Table 2.** Primary and Secondary Objectives Outcomes

Outcome	Overall (n = 254)	No OVP (n = 106)	OVP (n = 148)	*P* Value
CDI during ASCT hospitalization, No. (%)	18 (7)	12 (11)	6 (4)	.**03**
CDI during post-discharge follow-up, No. (%)	8 (3)	2 (2)	6 (4)	.47
Length of stay, d, median (range)	18 (12–37)	19 (12–37)	17 (12–37)	.**003**
Days of antibiotics during ASCT hospitalization, median (SD)	9 (5)	8 (3)	12 (5)	**<**.**001**
Time from admission to CDI detection, d, median (SD)	8 (4)	8.5 (3.8)	3.5 (4.2)	.14
CDI testing during ASCT hospitalization, No. (%)^a^	226 (89)	102 (96.2)	124 (83.8)	**<.01**
Time from OVP to CDI, d, median (range)	…	…	96.5 (21–173)	

Significant *P* values are displayed in bold type.

Abbreviations: ASCT, autologous stem cell transplant; CDI, *Clostridioides difficile* infection; OVP, oral vancomycin prophylaxis; SD, standard deviation.

^a^Counted as 1 test per patient, if patient had testing completed.

The use of OVP during ASCT hospitalization appeared to decrease risk of developing in-hospital CDI (hazard ratio [HR], 0.363 [95% confidence interval {CI}, .133–.995]; *P* = .049; Table 3). Median time from admission to positive *C difficile* among patients who developed CDI was 8.5 days (standard deviation [SD], 3.8 days) in the no-OVP group and 3.5 days (SD, 4.2 days) in the OVP group (overall range, 2–15 days; *P* = .14). In-hospital CDI testing rates were higher in the non-OVP group (96.2% vs 83.8%, *P* < .01; Table 2). No patients with a pre-ASCT history of CDI in either group (n = 9) developed *C difficile* recurrence during the ASCT admission.

**Table 3.** Cox Proportional Hazards Model for *Clostridioides difficile* Infection Development

Variable	HR (95% CI)	*P* Value
OVP, CDI during ASCT hospitalization^a^	0.363 (.133–.995)	.**049**
OVP, CDI in follow-up period^b^	1.949 (.393–9.658)	.41
Length of stay^c^	1.105 (1.002–1.196)	.**01**

Significant *P* values are displayed in bold type.

Abbreviations: ASCT, autologous stem cell transplant; CDI, *Clostridioides difficile* infection; CI, confidence interval; HR, hazard ratio; OVP, oral vancomycin prophylaxis.

^a^Risk of developing in-hospital CDI in patients who received OVP.

^b^Risk of developing CDI in the 180-day post-discharge follow-up period among patients who received OVP.

^c^Risk of developing CDI based on longer length of stay, regardless of patient group.

In the 180-day post-discharge follow-up period, 2 patients in the non-OVP group and 6 patients in the OVP group had a positive *C difficile* test (2% vs 4%, respectively, *P* = .47; Figure 1). Of those who received OVP, the median time to occurrence following OVP cessation was 96.5 days (range, 21–173 days). However, when censored for a competing risk of death, the use of OVP during ASCT did not correlate to increased risk of post-discharge CDI (HR, 1.949 [95% CI, .393–9.658]; *P* = .41; Table 3). No patients who had an in-hospital CDI diagnosis developed recurrence in the follow-up period. Overall, the sole episode of CDI recurrence observed in the study was 145 days post–OVP discontinuation in a patient with history of pre-ASCT CDI.

Patients who did not receive OVP experienced a longer median length of stay (19 vs 17 days, *P* = .003; Table 2). Additionally, the length of inpatient admission increased the risk of developing CDI (HR, 1.105 [95% CI, 1.002–1.196]; *P* = .01; Table 3). The median number of days of systemic antibiotic use during the ASCT admission was higher in the group that received OVP (12 vs 8 days, *P* < .001; Table 2). Patients received a median 32 OVP doses (16 days) in the prophylactic group (range, 6–100 doses). Subsequent to revised practice standards in 2019, 49.3% of the OVP population received PJP prophylaxis beginning at discharge or day +30, compared to zero in the non-OVP group. Of the 18 in-hospital CDI-positive patients, 17 (94%) were neutropenic (ANC <0.5 K/μL) at the time of CDI detection. Overall survival at day +180 from transplant was not different between OVP and no OVP (95.9% vs 96.2%, *P* = .91).

## DISCUSSION

The use of OVP limited the development of CDI during the ASCT period while not increasing risk for clinical CDI following OVP discontinuation. Testing rates were lower in the OVP group, which could be attributed to provider awareness of OVP use. However, our institution has consistently relied on standard staff-driven criteria for testing built within the EMR, thus limiting individualized provider bias. This finding may instead suggest lower rates of test-eligible patients due to OVP use.

Among all positive CDI cases, patients had undergone intensive conditioning with either BEAM or melphalan 200 mg/m^2^, which constituted most regimens in the study. These findings, while small, align with established CDI risk factors such as the use of myeloablative regimens [10]. Reduced-intensity regimens, usually defined as melphalan doses ≤140 mg/m^2^, may be less likely to be correlated with CDI; however, diarrhea and subsequent CDI testing are generally common among autologous transplant courses [11, 12].

Additionally, OVP appeared to offer a protective effect on the development of CDI against the known risk factor of systemic antibiotics, especially considering the significantly higher use of in-hospital antibiotics in the OVP group. This difference between groups may be correlated with a shift in practice for gram-negative prophylaxis. For the first 7 years of the study, systemic antimicrobial prophylaxis (almost always with fluoroquinolones) was administered only during the neutropenic period. In 2019, prophylaxis changed to begin earlier on the day of stem cell reinfusion and continued until ANC recovery. The lower incidence of CDI in OVP patients, despite increased systemic antibacterial use, correlates with the results of Van Hise and colleagues [13], who reported significantly lower CDI recurrence in patients receiving secondary OVP versus no OVP in concurrence with systemic antimicrobials (4.2% vs 26.6%, *P* < .001).

As noted by Johnson [14], the risks of OVP on normal gut microbiota may compromise its benefit. Our findings demonstrated no obvious correlation between OVP and subsequent development of clinical CDI in the months following OVP discontinuation. A larger CDI incidence may be able to elucidate significance. Given that 28.3% of the total study population also received PJP prophylaxis in the follow-up period, all of which had also received OVP, the lack of post-discharge CDI is notable as all of these patients had a known risk factor of continued antibiotic use. Of the OVP patients who developed CDI, the earliest incidence was at day 21, the end of the approximated “vulnerable period” as defined by Abujamel et al [8]. Half of these patients had CDI <2 months from OVP cessation while the other half occurred between 4 and 6 months. The timing of events extends the timeframe of reported post-OVP microbiota disruption patterns that state most occurrences are within 2 months [9]. Moreover, no in-hospital CDI patient developed post-treatment recurrence in the follow-up timeframe based on objective assessments of CDI testing only. There may be potential confounding factors during the follow-up period, such as the use of other systemic antibiotics or probiotics.

The time to in-hospital CDI detection between groups is noteworthy. The shorter median time to CDI in the OVP group may be related to preadmission colonization. All 5 patients who developed in-hospital CDI in 2018 or later had a PCR-positive but EIA toxin–negative result. Among that group, 80% received therapeutic dosing (4 times daily) of vancomycin. Furthermore, there were an additional 5 patients who developed out-of-hospital CDI in 2018 or later. Of these, only 1 patient was EIA toxin positive. Despite EIA negativity, treatment was based on clinician decision. However, it is possible that *C difficile* colonization was present but not the true cause of diarrhea in some patients [15]. We are unable to appropriately compare the effect of EIA detection and its influence on practices given the no-OVP group was limited to PCR alone.

Our findings of a shorter length of stay in the OVP group align with the results specific to the ASCT population by Shestovska et al [2]. Of note, the disproportion of transplant indications in our study may have contributed to shorter length of stay in the OVP-treated group. Patients more heavily conditioned with multiday BEAM regimens, of which the incidence was nearly half that in the OVP group, may inherently experience longer lengths of stay. These results correlate with existing data that conclude risk of CDI increases with longer length of stay [15].

The authors did not expect any effect of OVP use on transplant outcomes, but none was seen in a study of OVP use in allogeneic stem cell transplant patients [2, 4]. It is difficult to draw conclusions on correlation between CDI and survival as only 1 in-hospital CDI-positive patient in the entire population died before day +180.

A limitation of our research is the broad study time period, as other factors may have influenced rates of CDI. While CDI events were distributed throughout the study period, upon exploration of timing, it is noteworthy that among the no-OVP group, 7 (58%) of the CDI cases were clustered during periods of overlapping or consecutive admissions (eg, 2 CDI patients on the same floor during concurrent dates or a patient admitted to the same floor within 24 hours of a CDI-positive patient discharge).

There were no changes in infection prevention procedures or isolation policies during the study period. Ultraviolet light cleaning of rooms prior to each transplant admission was implemented prior to the study period. Although there was statistical significance, the similar rates of total CDI occurrence throughout the study period may beg the question of OVP preventing versus delaying the development of CDI. Given the overall low incidence of CDI rates in our population, it is challenging to draw further conclusions with respect to risk factors such as antibiotic use during ASCT admission or post-discharge, or prior CDI. We acknowledge that it is possible clinicians were biased toward less CDI testing when using OVP but propose that our nurse-driven standardized testing criteria dampen that affect. Another limitation is that neither VRE colonization nor gram-negative bacteremia incidence was assessed at any point in the study. Previous data have suggested no increase in VRE colonization with the use of OVP [4, 5]. However, data regarding gram-negative infection rates are mixed [2, 5]. The standard use of antibacterial prophylaxis during the neutropenic period was intended to mitigate gram-negative infection risk.

In summary, our data suggest a clinical benefit when OVP is utilized. Our institution plans to continue utilizing twice-daily OVP as primary prophylaxis. As many centers have begun to transition to outpatient ASCT, it is unknown if there remains a role for OVP when eliminating the key risk factor of hospitalization. We conclude that OVP is safe and effective with a reduction in clinical CDI episodes during the in-hospital ASCT period and does not increase delayed CDI post–OVP discontinuation.

## Notes

***Acknowledgments*.** We would like to thank all interdisciplinary members of the Aurora Malignant Hematology and Stem Cell Transplant Program as well as the Aurora Antimicrobial Stewardship Program for their support in this project.

***Author contributions.*** M. J. W., B. M., and J. G. performed data collection. S. K. and N. Z. performed data analysis. M. J. W., S. A., E. F., and H. R. collaborated to write the manuscript. B. P. B. conceived the original idea and provided edits. All authors critically reviewed and approved the final manuscript.

***Patient consent.*** This study does not include factors that necessitate patient consent. This project was approved by the Aurora Institutional Review Board and waived from oversight.

***Data availability.*** Data may be provided upon reasonable request.
